# Role of Cerebroplacental Ratio in Predicting Perinatal Outcome

**DOI:** 10.7759/cureus.54816

**Published:** 2024-02-24

**Authors:** Abhay Kumar, Anju Singh, Snigdha Kumari, S.C. Saha, Tulika Singh, Shiv Sajan Saini

**Affiliations:** 1 Obstetrics and Gynecology, Postgraduate Institute of Medical Education and Research, Chandigarh, IND; 2 Radiodiagnosis, Postgraduate Institute of Medical Education and Research, Chandigarh, IND; 3 Pediatric Medicine, Postgraduate Institute of Medical Education and Research, Chandigarh, IND

**Keywords:** middle cerebral artery doppler, umbilical artery doppler, perinatal outcome, high-risk pregnancy, cerebroplacental ratio

## Abstract

Objective

Doppler velocimetry provides a sensitive, non-invasive, and safe method of surveillance of fetal hemodynamics and fetomaternal circulation. Cerebroplacental ratio (CPR) is an indicator of placental function and fetal maladaptation to placental insufficiency. Cerebroplacental ratio (CPR) is becoming a significant indicator of unfavorable pregnancy outcomes, which has implications for the assessment of fetal well-being. This study aimed to determine the cut-off value of the cerebroplacental ratio (CPR) in appropriate for gestational age (AGA) fetuses in high-risk mothers to predict adverse perinatal outcomes. We also compared the efficacy of CPR, umbilical artery pulsatility index (UmA PI), and middle cerebral artery pulsatility index (MCA PI) for predicting adverse perinatal outcomes.

Design and setting

This was a prospective observational study conducted at the Postgraduate Institute of Medical Education and Research (PGIMER), Chandigarh, India.

Methods

A total of 100 women with singleton high-risk pregnancies were included in this prospective observational study. Obstetric ultrasound was performed at the time of recruitment, and fetal weight and CPR were noted. Based on fetal weight, patients were divided into AGA and fetal growth restriction (FGR) groups; CPR was measured; patients were followed up fortnightly; and outcomes were noted.

Main outcome

The effectiveness of CPR, UmA PI, and MCA PI for predicting poor perinatal outcomes and identifying the cut-off value of CPR in appropriate for gestational age (AGA) fetuses in high-risk mothers was assessed.

Result

The values of MCA PI, UmA PI, and CPR were statistically significant between AGA and FGR (p-value =.023, .002 and .0001), respectively. The cut-off value for CPR-detecting adverse outcomes in AGA was 1.49. It has sensitivity, specificity, positive predictive value, and negative predictive value of 67.5%, 68%, 71.69%, and 70.21%, respectively.

Conclusion

Cerebroplacental ratio (CPR) reflects both circulatory insufficiency of the placenta and adaptive changes of the middle cerebral artery, indicating an important non-invasive surveillance modality.

## Introduction

The growth of the fetus is one of the most important objectives in prenatal care. The growth of the fetus depends on many factors, such as uteroplacental function, nutrition, maternal disease, maternal cardiovascular function, altitude, smoking, illicit drug abuse, infections, aneuploidy, and genetic abnormalities.

Fetal growth restriction (FGR) is defined as a fetus not growing to its genetically predetermined potential. An estimated fetal weight less than the 10th centile is considered small for gestational age (SGA). The incidence of FGR is around 3-9% in the higher socioeconomic group, whereas in the lower socioeconomic group, it is 30% [1]. In India, the prevalence of low birth weight (LBW) was reported at 26%, while the proportion of FGR was found to be 56% [2]. As per the National Neonatal Perinatal Database of India, the reported incidence of FGR is 9.65% [3]. Fetal growth restriction (FGR) is more prevalent in resource-limited countries. In developed countries, 10% of term infants are SGA, and in resource-limited countries, it is 20% [4,5].

Fetal growth restriction is divided into early and late-onset FGR [6]. Early-onset FGR is diagnosed before 32 weeks of gestation by an abdominal circumference (AC) or estimated fetal weight (EFW) below the 3rd centile or by an AC or EFW less than the 10th centile, a uterine artery pulsatility index (PI) greater than the 95th centile, and/or an umbilical artery pulsatility index (UmA PI) greater than the 95th centile. Late-onset FGR is diagnosed after 32 weeks of gestation by an AC or EFW below the 3rd centile, or an EFW or AC below the 10th centile or its flattening across more than 2 centiles, and/or abnormal CPR or UmA PI.

Doppler velocimetry helps in the evaluation of the uterine and umbilical arteries to determine uteroplacental function, making it useful for measuring fetal growth. It also allows for the assessment of the middle cerebral artery (MCA) and the ductus venosus as the fetal cardiovascular adaptation progresses from hypoxia to acidemia. Doppler velocimetry provides a sensitive, non-invasive, and safe method of surveillance of fetal hemodynamics and fetomaternal circulation. Cerebroplacental ratio (CPR) is an indicator of placental function and fetal maladaptation to placental insufficiency. The CPR is determined as a ratio between the middle cerebral artery pulsatility index (MCA PI) and UmA PI. Reduced CPR may reflect a shift in brain blood flow distribution or a protective impact on the brain. The brain-sparing effect often manifests as cerebral vasodilatation to save blood supply to the brain in response to persistent hypoxia, an example of circulatory adaptation.

Cerebroplacental ratio (CPR) is becoming a significant indicator of unfavorable pregnancy outcomes, which has implications for the assessment of fetal well-being. The role of CPR is already known in the FGR fetus, but in AGA it is not known. Herein, we determine the cut-off value of cerebroplacental ratio (CPR) in appropriate for gestational age (AGA) fetuses in high-risk mothers for the prediction of adverse perinatal outcomes and compare the efficacy of CPR, umbilical artery pulsatility index (UmA PI), and middle cerebral artery pulsatility index (MCA PI) for predicting adverse perinatal outcomes.

## Materials and methods

After approval from the institute ethics committee, with reference number NK/7836/MD/325, 100 patients were enrolled in this prospective observational study between July 2021 and December 2022 at the Postgraduate Institute of Medical Education and Research, Chandigarh, in the Department of Obstetrics and Gynecology.

They were enrolled after obtaining written informed consent. The inclusion criteria include high-risk pregnancy with an age above 18 years, singleton pregnancy and a period of gestation between 32 and 36 weeks, diagnosed FGR or appropriate for gestational age, and willingness to participate. Exclusion criteria include multifetal pregnancy, congenital malformation in the fetus, elective cesarean section, antepartum stillbirth, malpresentation, and unwillingness to participate.

A high-risk pregnancy is defined as a pregnancy that is at increased risk of growth restriction, which includes risk factors like hypertensive disease (pre-eclampsia, chronic hypertension), chronic kidney disease (CKD), pre-gestational diabetes mellitus, autoimmune disease (systemic lupus erythematosus, antiphospholipid syndrome, autoimmune thyroid disorder), heart disease, assisted reproductive technique, heavy first trimester bleeding, previous stillbirth, diabetes with vascular disease, and maternal age greater than 35 years. All enrolled patients were evaluated with a detailed clinical history and physical examination. Her most recent menstrual cycle and an ultrasound performed in the first or early second trimester served as indicators of her gestational age. An obstetric ultrasound was performed at the time of recruitment to see the EFW, biophysical profile (BPP), and CPR. Hadlock's formula was used to determine EFW [7].

In the FGR group CPR, less than 1 was considered abnormal [8]. In FGR and AGA fetuses, CPR was done fortnightly and before induction of labor. The FGR fetus was monitored twice weekly by the biophysical profile (BPP) as per our institutional protocol. Pregnancies with abnormal CPR were not terminated, as there was no specific protocol for termination in the institute. Termination was done in both groups as per obstetrics/fetal indication.

The ultrasound machine used to calculate CPR was a real-time machine (Philips iU22) with a transducer frequency of 3.5-5.0 MHz. Doppler indices were calculated by built-in software programs in the machine. The examination was performed on the pregnant woman in a semi-recumbent position during relative fetal inactivity and apnea. This was done as end-diastolic flow (EDF) decreases with decreasing fetal heart rate and fetal breathing movements increase the variability in fetal Doppler. The UmA was seen in the middle of a free loop of the umbilical cord to avoid error, as EDF is higher near umbilical cord insertion into the fetal abdomen than near placental insertion. For MCA, a transverse image of the fetal head was obtained at the level of the sphenoid bones. Color flow imaging was used to display the circle of Willis. The MCA in the near field was insonated about 1cm distal to its origin from the internal carotid artery (ICA). By using this, the pulsatility index (PI) was calculated from the minimum of five consecutive waveforms on a frozen image. A series of three readings was taken from each artery to avoid errors. The CPR was calculated by dividing MCA PI and UmA PI.

Follow-up with study participants and termination of pregnancies were done as per institute protocols. The pregnancies were followed, and the final perinatal outcome of each was assessed by various indicators, which included operative vaginal delivery, cesarean section for fetal distress, umbilical cord PH <7.1, 5 min apgar less than 7, meconium aspiration, hypoglycemia <45 mg/dl, hyperbilirubinemia, admission to a neonatal unit (NNU) or neonatal intensive care unit (NICU), hypoxic-ischemic encephalopathy, birth weight less than the 10th centile, and intrauterine death (IUD). A p-value less than 0.05 is considered significant.

## Results

**Figure 1:**
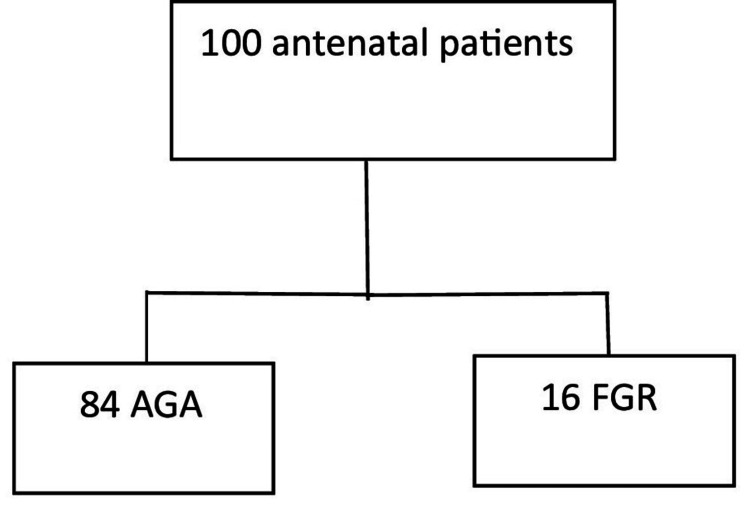
Recruitment of patients in the study

This was a prospective observational study conducted between July 2021 and December 2022. In this study, we recruited 100 patients with high-risk pregnancies and further divided them into FGR (fetal growth restricted) and AGA (appropriate for gestational age) (Figure 1).

Hypertensive disease in pregnancy (n = 45) (45%) and type 2 diabetes (n = 23) (23%) contribute to most of the patients in the study. The CPR was calculated, and adverse outcomes were noted. The mean gestational age of recruitment AGA and FGR were comparable (35.01+/-1.19 and 34.36+/-1.66, p-value =.064). The baseline parameters, including age, socioeconomic status, parity, body mass index (BMI), and conception, were comparable except for BMI (p-value =.041) (Table 1).

**Table 1:** Demographic characteristics of AGA & FGR fetuses spon: spontaneous; primi: primigravida; multi: multigravida; BMI: body mass index; AGA: appropriate for gestational age; FGR: fetal growth restriction

		AGA (n=84), N(%)	FGR(n=16), N(%)	p-value
Age (in years)	<=35	66 (78.6%)	15 (93.8%)	0.156
	>=35	18 (21.4%)	1 (6.2%)	
Socioeconomic status	Lower middle	55 (65.5%)	14 (87.5%)	0.081
	Upper middle	29 (34.5%)	2 (12.5%)	
BMI (kg/m2)	Normal	66 (78.6%)	16 (100%)	.041*
	Overweight	18 (21.4%)	0 (0.0%)	
Conception	Spon	77 (91.7%)	14 (87.5%)	0.594
	Assisted	7 (8.3%)	2 (12.5%)	
Parity	Primi	50 (59.5%)	11 (68.8%)	.488
	Multi	34 (40.5%)	5 (31.2%)	

The per abdomen examination and symphysis fundal height (SFH) was measured at the time of recruitment, and the difference was statistically significant (p-value =.0001 and .0001), weight at recruitment between the two groups is 2.29 +/-.34 kg and 1.61 +/-.23 kg, and the difference was statistically significant (p-value =.0001) (Table 2).

**Table 2:** Characteristics of patients at recruitment POG: period of gestation; SFH: symphysis fundal height; AGA: appropriate for gestational age; FGR: fetal growth restriction

	AGA (n-84), Mean ± SD	FGR (n-16), Mean ± SD	p-value
POG (weeks)	35.01±1.19	34.36±1.66	.064
Weight (kg)	2.29±0.34	1.61±0.23	.0001*
Fundal height (weeks)	35.12±1.10	31.69±2.52	.0001*
SFH (cm)	34.86±1.18	31.81±2.04	.0001*

The Doppler indices were measured at the time of recruitment. The values of MCA PI, UmA PI, and CPR were noted. There was a statistically significant difference between AGA and FGR (p-values =.023,.002, and.0001) (Table 3).

**Table 3:** Doppler indices at recruitment AGA: appropriate for gestational age; FGR: fetal growth restriction; CPR: cerebroplacental ratio; MCA PI: middle cerebral artery pulsatility index

	AGA (n-84), Mean ± SD	FGR (n-16), Mean ± SD	p-value
Umbilical artery PI	1.09±0.42	1.64±1.24	.002*
MCA PI	1.59±0.40	1.35±0.25	.023*
CPR	1.55±0.52	0.98±0.33	.0001*
Right uterine artery PI	0.87±0.34	1.02±0.42	.117
Left uterine artery PI	0.83±0.22	0.91±0.27	.188

All patients in the FGR group were induced, and in AGA, 76.2% were induced, and the rest underwent spontaneous labor. The gestational age at delivery was comparable in the two groups (p-value =.117). In FGR, 43.8% of babies were born via cesarean section (CS), whereas in AGA, it was 19%. The proportion of hyperbilirubinemia (56.3%) and NICU admissions (6.3%) was higher in the FGR group. There was one intrauterine fetal death in a patient with pre-eclampsia without severe features in the FGR group. The overall proportion of adverse events is higher in FGR than in AGA (p-value =.0001) (Table 4).

**Table 4:** Comparisons of adverse neonatal outcomes in AGA and FGR NICU: neonatal intensive care unit; CS: cesarean section; IUD: intrauterine fetal death; AGA: appropriate for gestational age; FGR: fetal growth restriction

		AGA (n-84),N(%)		FGR (n-16),N(%)		p-value
Hypoglycemia <45mg/dl	Yes	6	7.1%	1	6.3%	0.898
	No	78	92.9%	15	93.8%	
Hyperbilirubinemia(>5mg/dl)	Yes	21	25.0%	9	56.3%	.012*
	No	63	75.0%	7	43.8%	
Admission to NICU	Yes	0	0.0%	1	6.3%	.021*
	No	84	100.0%	15	93.8%	
Birth weight <10^th^ centile	Yes	13	15.5%	15	93.8%	.0001*
	No	71	84.5%	1	6.3%	
CS for fetal distress	Yes	16	19%	7	43.8%	.031*
	No	68	81%	9	56.3%	
IUD	Yes	0	0.0%	1	6.3%	0.3513
	No	84	100.0%	15	93.8%	

**Figure 2:**
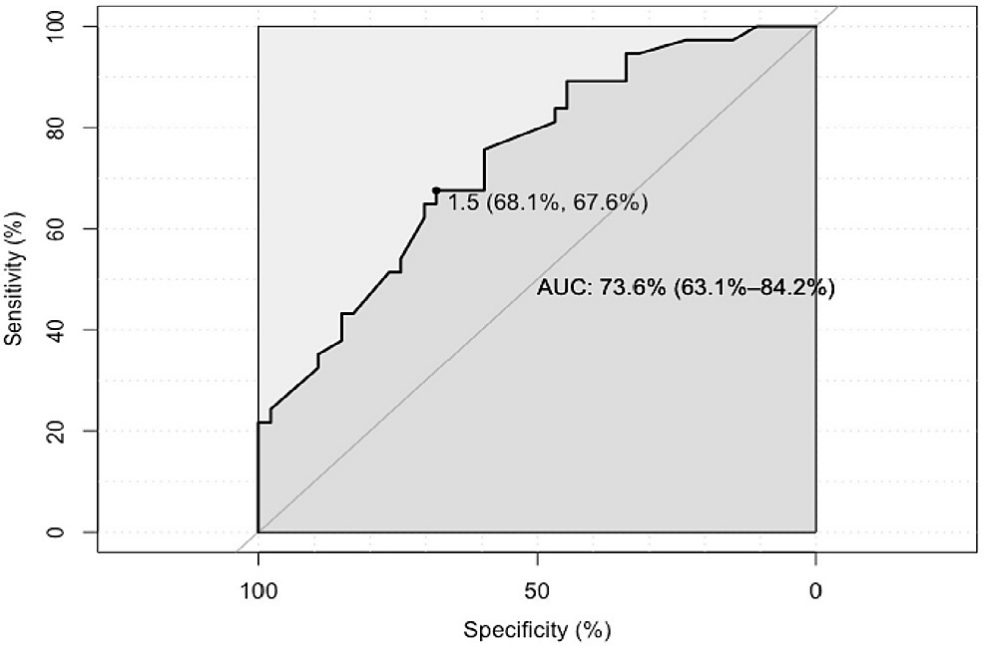
Cut-off value of CPR in AGA in predicting adverse outcomes AGA: appropriate for gestational age; CPR: cerebroplacental ratio

The cut-off value for CPR-detecting adverse outcomes in AGA was 1.49. It has a sensitivity and specificity of 67.6% and 68% (Figure 2). With a positive predictive value and a negative predictive value of 71.69% and 70.21%, respectively.

**Figure 3:**
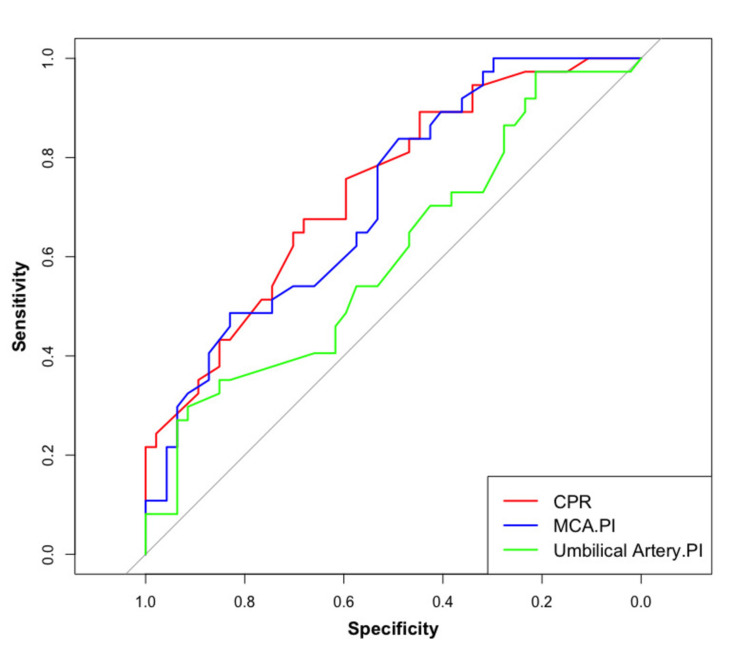
Comparison of CPR, MCA PI, and UmA PI in the AGA fetus in predicting adverse outcomes CPR: cerebroplacental ratio; MCA PI: middle cerebral artery pulsatility index; UmA PI: umbilical artery pulsatility index

The CPR predicts a better adverse outcome than MCA PI and UmA PI in AGA fetuses (Figure 3).

**Figure 4:**
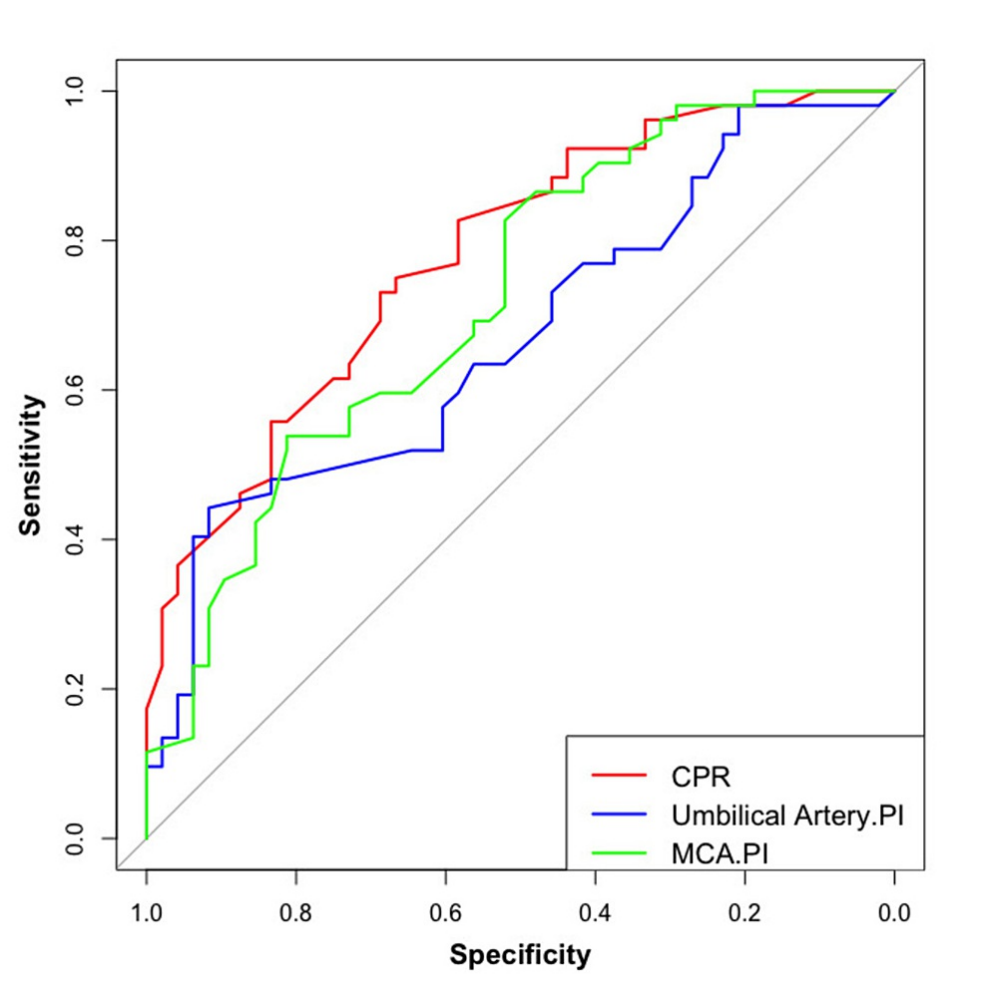
Overall comparison of CPR, MCA PI, and UmA PI in predicting adverse outcomes. CPR: cerebroplacental ratio; MCA PI: middle cerebral artery pulsatility index; UmA PI: umbilical artery pulsatility index

The overall performance of CPR in predicting adverse outcomes is better than MCA PI in both AGA and FGR fetuses (Figure 4).

## Discussion

Cerebroplacental ratio (CPR) has become an important tool for detecting adverse perinatal outcomes. It is a well-known fact that FGR fetuses are at risk of adverse perinatal outcomes; however, the successful identification of AGA fetuses, which are at risk of adverse outcomes, is very challenging and is dependent on various factors. The CPR< 1 is well known to be associated with poor perinatal outcomes in FGR fetuses, but there is no specific cut-off value in AGA fetuses. The mean CPR value in AGA was 1.55 and in FGR was 0.98, and the difference was statistically significant (p-value =.0001). The proportion of adverse outcomes was higher in FGR when compared to AGA. The cerebral-umbilical Doppler ratio was a better predictor of adverse perinatal outcomes [9]. Most of the studies selected a predetermined cut-off value of 1 in both AGA and FGR for evaluating unfavorable outcomes. In this study, a cut-off value of 1.0 was selected in FGR fetuses, whereas in AGA fetuses, Doppler indices were noted, and the CPR cut-off value was investigated and found to be 1.49, which had a sensitivity and specificity of 68% and 67.5% in detecting adverse outcomes, with positive and negative predictive values of 71.6% and 70.2%. According to the Standard Criteria, CPR's AUC of 0.73 is acceptable as a predictor. Bahado et al. [10] also predicted that abnormal CPR was associated with increased cesarean delivery and NICU admission, but it was not consistent with our study because all the pregnancies were terminated before 34 weeks of gestation in their study. However, in our study, the mean age of delivery in AGA was 37.4 weeks, and in FGR it was 36.96 weeks. On average, most of the fetuses were delivered at 36-37 weeks. Like Cruz Martinez et al. [11], we also found that CPR is more sensitive than MCA PI in the prediction of adverse outcomes. In our study, there is a significant correlation between CPR and adverse outcomes, which is consistent with a study by Fiolna et al. in which low CPR, measured within 24 hours before induction of labor, is associated with an increased risk of a cesarean section for fetal distress and adverse neonatal outcomes [12].

Fetal growth restriction that goes undetected increases the likelihood of several unfavorable perinatal outcomes. A fetus that is believed to be of normal size for gestation but is actually growth-restricted has an eight-fold greater risk of stillbirth compared to a normally developed pregnancy [13]. This was an important finding that was particularly relevant to our study. We measured the CPR in AGA and FGR fetuses. The proportion of adverse outcomes is higher in FGR, but there is also increased risk in AGA fetuses, as shown in our study (93.8% vs. 44%). According to other studies, it may be possible to detect at-risk fetuses by monitoring fetal weight and circulatory assessment during the third trimester. Fetal growth restriction that goes undetected increases the likelihood of several unfavorable perinatal outcomes.

The premise of this study is that, if impaired placentation is the cause of adverse outcomes, prenatal care should concentrate on identifying hypoxemic fetuses rather than just small ones, and screening should concentrate on identifying pregnancies with low CPR rather than just those with low estimated fetal weights. The CPR can help in risk-stratifying pregnancies promptly, especially before labor, which guides decision-making in a non-emergent situation regarding the timing and mode of delivery. It helps in the timely referral of patients with low CPR to a tertiary care center for better maternal and fetal surveillance and, lastly, termination with better maternal and neonatal facilities. It is likely to result in improved maternal satisfaction and the birth experience. Cerebroplacental ratio (CPR) should be incorporated into routine third-trimester antepartum fetal monitoring; it helps in the detection of hypoxemic fetuses that are at risk of adverse perinatal outcomes. It further helps in better surveillance of the fetus and pregnancy outcomes. Pregnancies that are at high risk of adverse outcomes, such as cesarean delivery for fetal distress, etc., and adverse neonatal outcomes, should be better managed by elective CS. Emergency cesarean delivery often carries more complications and parental anxiety, and it often occurs out of hours when staff is inadequate. It also helps in identifying the need for intensive fetal monitoring during labor. Given our results and those of others, it is reasonable to add CPR to routine antepartum fetal monitoring in the third trimester. The limitations of the study were that the sample size of this study was less (100 participants). The fetus that underwent preterm labor was not excluded; hence, some adverse outcomes were exaggerated.

## Conclusions

 In fetal growth-restricted fetuses, CPR reflects both circulatory insufficiency of the placenta as well as adaptive changes of the middle cerebral artery, so it indicates an important non-invasive modality for surveillance. In high-risk AGA fetuses, CPR is also helpful in detecting fetuses that are at risk of adverse outcomes, so it helps in the early identification, monitoring, and overall management of fetuses. Hence, Doppler ultrasound, especially CPR, should be an integral part of routine third-trimester ultrasound examinations in high-risk AGA pregnancies.

## References

[1] Lee AC, Kozuki N, Cousens S (2017). Estimates of burden and consequences of infants born small for gestational age in low and middle income countries with INTERGROWTH-21(st) standard: analysis of CHERG datasets. BMJ.

[2] Saleem T, Sajjad N, Fatima S, Habib N, Ali SR, Qadir M (2011). Intrauterine growth retardation--small events, big consequences. Ital J Pediatr.

[3] Fanaroff AA, Hack M, Walsh MC (2003). The NICHD neonatal research network: changes in practice and outcomes during the first 15 years. Seminars in perinatology.

[4] Anderson MS, Hay WW (1999). Intrauterine Growth Restriction and the Small-for-Gestational Age Infant.

[5] de Onis M, Blössner M, Villar J (1998). Levels and patterns of intrauterine growth retardation in developing countries. Eur J Clin Nutr.

[6] Gordijn SJ, Beune IM, Thilaganathan B (2016). Consensus definition of fetal growth restriction: a Delphi procedure. Ultrasound Obstet Gynecol.

[7] DeVore GR (2015). The importance of the cerebroplacental ratio in the evaluation of fetal well-being in SGA and AGA fetuses. Am J Obstet Gynecol.

[8] Burd I, Srinivas S, Paré E, Dharan V, Wang E (2009). Is sonographic assessment of fetal weight influenced by formula selection?. J Ultrasound Med.

[9] Gramellini D, Folli MC, Raboni S, Vadora E, Merialdi A (1992). Cerebral-umbilical Doppler ratio as a predictor of adverse perinatal outcome. Obstet Gynecol.

[10] Bahado-Singh RO, Kovanci E, Jeffres A, Oz U, Deren O, Copel J, Mari G (1999). The Doppler cerebroplacental ratio and perinatal outcome in intrauterine growth restriction. American journal of obstetrics and gynecology.

[11] Cruz-Martínez R, Figueras F, Hernandez-Andrade E, Oros D, Gratacos E (2011). Fetal brain Doppler to predict cesarean delivery for nonreassuring fetal status in term small-for-gestational-age fetuses. Obstet Gynecol.

[12] Fiolna M, Kostiv V, Anthoulakis C, Akolekar R, Nicolaides KH (2019). Prediction of adverse perinatal outcome by cerebroplacental ratio in women undergoing induction of labor. Ultrasound Obstet Gynecol.

[13] Gardosi J, Madurasinghe V, Williams M, Malik A, Francis A (2013). Maternal and fetal risk factors for stillbirth: population based study. BMJ.

